# Mesenchymal Stem/Stromal Cells Reverse Adipose Tissue Inflammation in Pigs with Metabolic Syndrome and Renovascular Hypertension

**DOI:** 10.3390/cells14010040

**Published:** 2025-01-02

**Authors:** Alexander B. C. Krueger, Xiangyang Zhu, Sarosh Siddiqi, Emma C. Whitehead, Hui Tang, Kyra L. Jordan, Amir Lerman, Lilach O. Lerman

**Affiliations:** 1 Division of Nephrology & Hypertension, Mayo Clinic, 200 1st Street SW, Rochester, MN 55905, USA; krueger.alexander@mayo.edu (A.B.C.K.); zhu.xiangyang@mayo.edu (X.Z.); emma.whitehead@duke.edu (E.C.W.); tang.hui@mayo.edu (H.T.); jordan.kyra@mayo.edu (K.L.J.); 2 Department of Cardiovascular Diseases, Mayo Clinic, 200 1st Street SW, Rochester, MN 55905, USA; lerman.amir@mayo.edu

**Keywords:** metabolic syndrome, renal artery stenosis, mesenchymal stem cells, inflammation

## Abstract

Metabolic syndrome (MetS) is associated with low-grade inflammation, which can be exacerbated by renal artery stenosis (RAS) and renovascular hypertension, potentially worsening outcomes through pro-inflammatory cytokines. This study investigated whether mesenchymal stem/stromal cells (MSCs) could reduce fat inflammation in pigs with MetS and RAS. Twenty-four pigs were divided into Lean (control), MetS, MetS + RAS, and MetS + RAS + MSCs. In the MSC-treated group, autologous adipose-derived MSCs (10^7^ cells) were injected into the renal artery six weeks after RAS induction. After four weeks, fat volumes and inflammatory markers were assessed. MSC treatment reduced levels of pro-inflammatory cytokines (MCP-1, TNF-a, IL-6) in the renal vein blood and in perirenal fat. The MSCs also decreased fat fibrosis, restored adipocyte size, and altered adipogenesis-related gene expression, particularly in the perirenal fat. These effects were less pronounced in subcutaneous fat. The MSC therapy attenuated fat inflammation and improved metabolic outcomes in pigs with MetS + RAS, suggesting that adipose-derived MSCs may offer a promising therapeutic approach for metabolic disorders.

## 1. Introduction

Metabolic syndrome (MetS) is a proinflammatory state characterized by central obesity, hypertension, dyslipidemia, and insulin resistance [1]. Together, these conditions significantly increase the risk of developing type-2 diabetes, heart disease, and stroke. In MetS, dysfunctional adipocytes release inflammatory adipokines that promote and sustain fat tissue inflammation, which is further entrenched by infiltrating immune cells that produce cytokines and chemokines [2]. Moreover, perivascular adipose tissue regulates vascular tone, and in obesity, its secretory profile shifts to favor vasoconstriction [3].

Fat tissue remodeling in MetS occurs chiefly through hyperplasia and hypertrophy. Postnatal adipose tissue expansion occurs through hyperplasia, primarily in subcutaneous fat in young individuals, and through hypertrophy, characterized by increased adipocyte size and fat content due to an energy surplus. Adipocyte hypertrophy is associated with inflammation and obesity-related morbidities [4]. The early phase of clonal expansion during preadipocyte hypertrophy is regulated by C/EBP (CCAAT/enhancer-binding protein)-a and -b, transcription factors that play a critical role in upregulating the nuclear receptor PPARy (Peroxisome Proliferator-Activated Receptor-gamma) [4]. PPARy decreases insulin resistance, reduces inflammation, and differentiates preadipocytes into mature adipocytes [5].

Hypertrophied adipocytes play a crucial role in proinflammatory cascades by releasing monocyte chemoattractant protein-1 (MCP-1), tumor necrosis factor-alpha (TNF-a), interleukin-6 (IL-6), and other proinflammatory cytokines/chemokines [6]. This can promote maladaptive and feed-forward fat tissue remodeling, including adipocyte hypertrophy [7], increased fibrosis, insulin resistance, and ultimately, MetS. This pro-inflammatory, diabetogenic, and atherogenic profile also contributes to secondary organ damage to the liver, brain, endothelium, and other organs [8], as well as hypertension.

Fat surplus can accumulate in several depots [9], with distinct implications for health. Subcutaneous fat primarily serves as an energy reserve and is generally associated with a low independent risk for metabolic complications [10]. In contrast, visceral fat encases internal organs in the abdominal cavity, including the kidney. It is more metabolically active than subcutaneous fat, releasing inflammatory cytokines and free fatty acids, and is linked to a risk of MetS [10]. Specifically, peri-renal fat is an independent risk factor for loss of renal function [11] and regulates renal vascular function [12]. Obesity also damages the kidneys directly by inducing hyperfiltration [13] and activating the renin–angiotensin–aldosterone system (RAAS), which may lead to kidney fibrosis and microvascular remodeling.

MetS frequently coexists with atherosclerosis and vascular diseases such as renal artery stenosis (RAS), which can further exacerbate the outcomes of MetS by triggering pro-inflammatory cytokine release [14,15]. RAS is characterized by ischemia, fibrosis, and prominent inflammation within the post-stenotic kidney, including upregulation of MCP-1, TNF-a, and IL-6, which instigates inflammation in the systemic circulation [16]. Additionally, RAS activates the RAAS, impairing kidney function and structure and leading to the development of renovascular hypertension (RVH). Hypertension in turn can induce fat remodeling via angiotensin-II receptors expressed in adipocytes [17,18]. Thus, the coexistence of these conditions can be particularly harmful for both the kidney and fat tissue remodeling. Given the rising prevalence of MetS, as well as RAS and their associated morbidity and mortality, there is a critical need for new therapies to address these harmful effects.

MSCs are multipotent reparative cells with regenerative and immunomodulatory potential [19]. They have shown promise in treating MetS [20] and have successfully reduced kidney injury and functional decline in pigs with RAS and RVH [21]. MSCs target central pathogenic mechanisms activated by both RAS and MetS, such as inflammation, fibrosis, microvascular loss, and adverse tissue remodeling, by reducing the release of inflammatory cytokines through key vectors, such as the anti-inflammatory protein tumor necrosis factor-stimulated gene-6 (TSG-6) [22]. This reduced inflammation may in turn blunt insulin resistance, endothelial dysfunction, RAAS activation, and ultimately, renal damage [23].

Practical advantages of allogeneic MSCs include the ability to harvest them from various tissues, their minimal provocation of immune response, and the avoidance of ethical issues associated with embryonic cells. Thus, MSCs are promising candidates for treating metabolic and inflammatory disorders.

Adipose tissue-derived MSCs have shown the capability to reduce inflammation and RAAS activation [24], yet their ability to attenuate fat tissue remodeling in MetS with concurrent RVH has not been explored. This study, therefore, tested the hypothesis that intrarenal delivery of MSCs would decrease the accumulation of inflammatory mediators and the degree of fat tissue remodeling in the perirenal and subcutaneous fat of pigs with MetS + RAS.

## 2. Materials and Methods

### 2.1. Study Design and Animal Preparation

**Figure 1:**
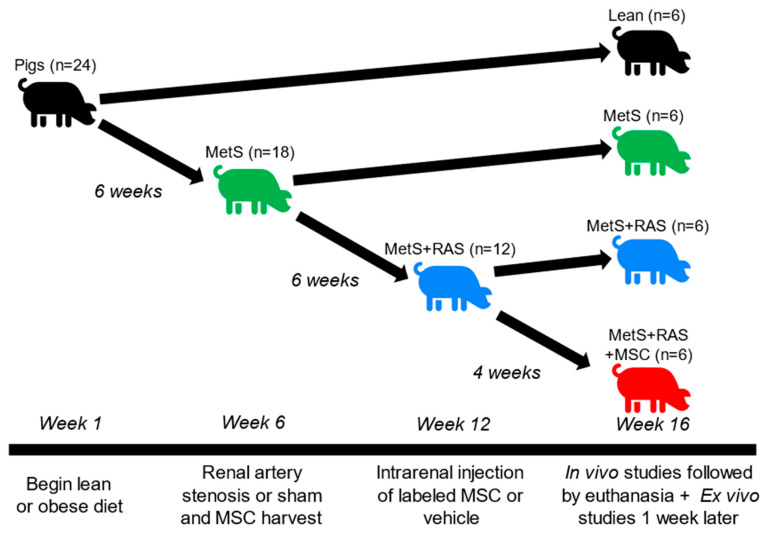
Schematic of the experimental protocol and treatment timeline.

Domestic female pigs (*n* = 24, 50–60 Kg) were studied during sixteen weeks of observation and randomly assigned to four groups: Lean, MetS, MetS + RAS, and Mets + RAS + MSC (*n* = 6 each). Eighteen pigs were fed with a MetS-inducing diet. Of these, after six weeks of diet, twelve pigs also underwent surgical induction of RAS and subcutaneous fat tissue collection for MSC harvest. Another six weeks later, six of these MetS + RAS pigs were treated with intrarenal delivery of autologous adipose tissue-derived MSCs (Figure 1). Perirenal and visceral fat volume was determined in vivo in all pigs four weeks later using computed tomography (CT). All procedures were approved by the Mayo Clinic Institutional Animal Care and Use Committee on 13 January 2015.

### 2.2. Specific Methods

At baseline, eighteen pigs with MetS were fed a high cholesterol/carbohydrate diet (5B4L; containing 16.1% protein, 43.0% ether-extract fat, and 40.8% carbohydrates; Purina Test Diet, Purina Animal Nutrition LLC, Richmond, IN, USA). Meanwhile, six other pigs were given standard pig chow. After six weeks (Figure 1), the pigs were anesthetized, and RAS was induced in twelve pigs by placing a local irritant coil in the main renal artery [25]. The remaining pigs underwent sham renal angiography. Fat tissue was collected via an abdominal subcutaneous biopsy to isolate autologous MSCs [26]. Six weeks later, six MetS + RAS pigs received an injection of 1 × 10^7^ MSCs suspended in 10 mL of phosphate-buffered saline (PBS) into the stenotic renal artery over 5 min, using a 5F catheter positioned proximal to the stenosis. This MSC dose was selected based on several prior studies that demonstrated its efficacy using this regimen, including in a RAS pig model [27]. The control groups received 10 mL of PBS alone.

Four weeks later, the pigs were anesthetized. The mean arterial pressure (MAP) was monitored during CT studies using an arterial catheter, and blood samples were taken from a systemic and stenotic kidney vein for measuring biochemical parameters. The pigs were then euthanized with an intravenous bolus of Fatal-Plus (15 mL, Vortech Pharmaceuticals, Dearborn, MI, USA), and both perirenal and subcutaneous fat was collected for histological and molecular assays (Figure 1).

MSCs were isolated from 5–10 g of adipose tissue using collagenase, following a standard protocol [26]. The cells were cultured in advanced MEM medium (Gibco/Invitrogen, Grand Island, NY, USA) supplemented with 5% platelet lysate (Mayo Clinic Transfusion Medicine, Rochester, MN, USA) at 37 °C in a 5% CO_2_ incubator. For later characterization, the cells were stored in a recovery medium at −80 °C. MSCs were identified by the expression of common markers (CD44, CD90, and CD105). Their ability to differentiate into adipocytes, chondrocytes, and osteocytes was also assessed, as described in previous studies [28].

### 2.3. Systemic and Post-Stenotic Renal Vein Blood Analysis

Systemic and renal inflammation was evaluated by measuring MCP-1 and TNF-a levels in the systemic and renal veins by ELISA (BMS281 and KHC3011, respectively, ThermoFisher Scientific, Waltham, MA, USA). Plasma Renin Activity (PRA) was measured using a renin assay (Sigma-Aldrich, St. Louis, MO, USA, Cat#MAK157) and serum creatinine (SCr) using a kit (Arbor Assays, Ann Arbor, MI, USA, Cat#KB02). Systemic blood was also utilized for fasting glucose, insulin, and lipid profile measurements. Fasting insulin and glucose levels were used to calculate the Homeostatic Model Assessment for Insulin Resistance (HOMA-IR) [29].

### 2.4. Adipose Tissue Volume

Non-contrast axial CT scans were taken of each pig to calculate visceral and subcutaneous fat volume. For visceral fat analysis, a region of interest (ROI) was placed on the visceral fat (at the level of renal hilum), and the volume was calculated using Analyze^®^ Version 12.0 (Mayo Clinic, Rochester, MN, USA) software’s sampling options. The fat tissue density was used to automatically threshold it in the entire image and aggregate all pixels within the visceral fat ROI to determine the total volume [30]. The same procedure was applied to the subcutaneous fat. The percentage of visceral and subcutaneous fat relative to total fat was then calculated. Representative images were created using 3D Slicer software (The Slicer Community, Boston, MA, USA).

### 2.5. Adipose Tissue Remodeling

Visceral and subcutaneous fat fibrosis and adipocyte size were assessed in trichrome-stained (blue) and eosin counterstained (pink) 5 µm sections at 10× magnification. The images were analyzed using ImageJ (NIH, Annapolis, MD, USA) to calculate % field area positively blue-stained for fibrosis and adipocyte size [31].

### 2.6. Adipose Tissue Inflammatory Markers

Perirenal and subcutaneous fat was immunofluorescently stained for MCP-1 (cat#ab9669, Abcam, Cambridge, UK, 1:100), TNF-a (Cat#SC-133193, Santa Cruz, Dallas, TX, USA, 1:100), and IL-6 (cat#ab6672, Abcam, 1:500). Images were taken at 40× for one slide per pig from 10 fields, and the % of positively stained area was calculated using ImageJ.

To assess metabolic and inflammatory gene expression, frozen adipose tissue (10 mg) from each depot was homogenized in 400 µL of ice-cold lysis buffer from the mirVana PARIS total RNA isolation kit (ThermoFisher, Cat# AM1556). Total RNA was isolated, and its concentration was measured (ThermoFischer NanoDrop Spectrophotometer). A 50 µL portion of the RNA samples was treated with DNase (ThermoFisher, Cat#AM1906). First-strand cDNA was synthesized from 800 ng of total RNA (SuperScript VILO Master Mix, ThermoFisher, Cat#11755-050). Quantitative PCR was performed using ThermoFischer TaqMan assays, with 16 ng of cDNA in each reaction. The following primers from ThermoFisher Scientific were used: MCP-1 (ss03394377), TNF-a (ss03391318), IL-6 (ss07308316), C/EBPa (ss03373315), C/EBPb (ss03375347), PPARy (ss03394829), and TSG-6 (ss04246163). GAPDH (Cat# ss03375629) served as the reference control. Negative controls (no cDNA) were included in each run. PCR analysis was conducted on an Applied Biosystems QuantStudio-7 PCR system with the following conditions: 50 °C for 2 min, 95 °C for 10 min, and 40 cycles of 95 °C for 15 s and 60 °C for 1 min. The fold changes in gene expression for each target gene were calculated relative to the control group using the 2^−ΔΔCT^ method.

### 2.7. Statistical Analysis

A sample size of *n* = 6 for each group was selected based on previous studies demonstrating the detectability of statistically significant differences in the MetS and RAS kidneys with 6 subjects [32]. Data were analyzed using GraphPad Prism-9 (GraphPad Software, San Diego, CA, USA). The results were tested for normality using the Shapiro–Wilk test, and the normally distributed variables are expressed as mean ± standard deviation. Comparisons within groups were performed using paired Student’s *t*-test and a one-way ANOVA was used for multiple groups. A statistically significant difference was considered for *p* ≤ 0.05.

## 3. Results

### 3.1. Systemic Characteristics

At 16 weeks, the MetS diet resulted in significantly heavier MetS and MetS + RAS pigs compared to the Lean pigs (Table 1). The MetS + RAS + MSC pigs were smaller than the MetS pigs, but their weight was not significantly different from either Lean or MetS + RAS groups. The Lean group had a statistically significant lower total cholesterol compared to all other groups. However, there was no statistically significant difference in fasting glucose or insulin levels among the groups. The HOMA-IR in the MetS + RAS + MSC group was not significantly different relative to the Lean group but was significantly lower than in the MetS + RAS pigs. SCr was not different among the four pig groups, whereas all the experimental groups had significantly higher PRA than the Lean group (Table 1). The MAP was higher in the MetS and MetS + RAS group compared to Lean group, but in the MetS + RAS + MSC group, the MAP was not significantly different from any of the other groups (Table 1).

### 3.2. Intrarenally Injected MSCs Decrease Inflammation

The MCP-1 levels in the post-stenotic kidney vein were elevated in the MetS + RAS group relative to the Lean and MetS groups but trended lower in the MetS + RAS + MSC group relative to the MetS + RAS group (*p* = 0.076) (Table 1). The systemic MCP-1 levels were similarly elevated in all experimental groups relative to the Lean group. The TNF-a levels in the post-stenotic kidney vein trended higher in the MetS + RAS pigs vs. the Lean pigs (*p* = 0.063) and, again, tended to be lower in the MetS + RAS + MSC renal vein vs. MetS + RAS (*p* = 0.079) (Table 1). The systemic TNF-a levels were not statistically different among all groups.

### 3.3. MSCs Attenuate Visceral Adipose Accumulation

**Figure 2:**
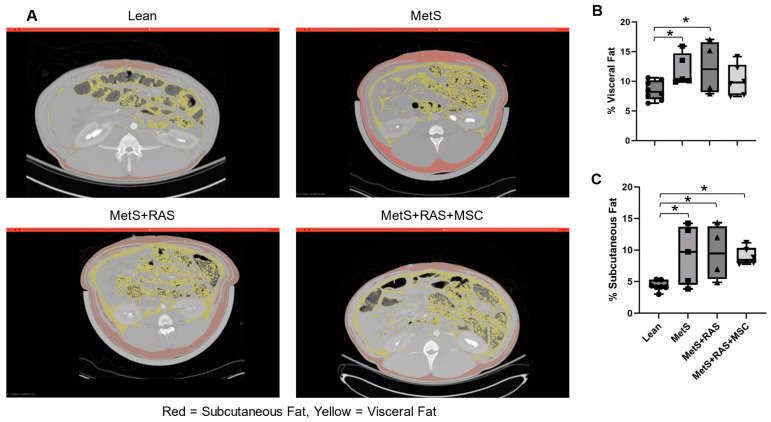
MSCs downregulate visceral adipose tissue accumulation. (**A**) Abdominal CT axial images with post-imaging processing highlighting subcutaneous (red) and visceral (yellow) fat. (**B**) Visceral fat fraction was augmented in MetS and MetS + RAS, but not in MetS + RAS + MSC. (**C**) The subcutaneous fat fraction was increased in all 3 experimental groups relative to Lean. * *p* < 0.05 vs. Lean. MetS, metabolic syndrome; RAS, renal artery stenosis; MSC, mesenchymal stem/stromal cells.

Abdominal CT scans revealed statistically significant elevations in subcutaneous adipose volume in all experimental groups relative to Lean (Figure 2A,C). However, the visceral fat volume was elevated only in the MetS and MetS + RAS groups, but not in the MetS + RAS + MSC group relative to Lean (Figure 2A,B). This suggests that visceral fat accumulation was attenuated by the MSCs.

### 3.4. MSCs Reduce Perirenal Fat Remodeling

**Figure 3:**
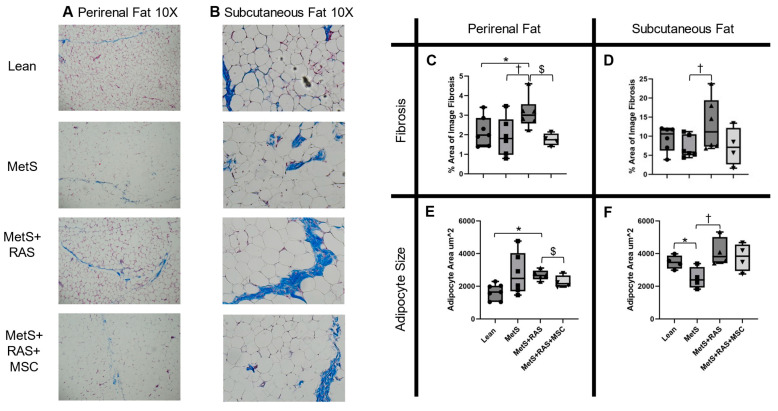
MSCs attenuate adipose tissue fibrosis and hypertrophy. Representative images of trichrome-stained (blue) perirenal (**A**) and subcutaneous (**B**) fat (10× magnification). Pink: eosin counterstain. (**C**) Perirenal fat fibrosis increased in MetS + RAS but was attenuated by the delivery of MSCs. (**D**) Subcutaneous fat fibrosis was only higher in MetS + RAS vs. MetS. (**E**) Adipocyte cross-sectional area was higher in the perirenal fat of MetS + RAS vs. Lean, suggesting hypertrophy, but decreased in MetS + RAS + MSC. (**F**) In the subcutaneous fat, MetS had smaller adipocytes compared to Lean and MetS + RAS. * *p* < 0.05 vs. Lean; ^†^ *p* < 0.05 vs. MetS; ^$^ *p* < 0.05 vs. MetS + RAS. MetS, metabolic syndrome; RAS, renal artery stenosis; MSC, mesenchymal stem/stromal cells.

Trichrome staining of perirenal adipose tissue revealed elevated fibrosis in the MetS + RAS pigs relative to both the Lean and MetS groups, which was attenuated by the MSCs (Figure 3A,C). Subcutaneous fat showed elevated fibrosis in the MetS + RAS groups vs. the MetS groups, which were unaffected by the MSCs (Figure 3B,D). Thus, the MSCs attenuated fibrosis in the perirenal fat to a greater extent than in subcutaneous fat.

Trichrome staining of perirenal fat also revealed increased adipocyte size in the MetS + RAS pigs relative to the Lean pigs, with a small but significant decrease observed in the MetS + RAS + MSC group vs. the MetS + RAS group (Figure 3A,E). In the subcutaneous fat, adipocyte size was decreased in the MetS group relative to the Lean and the MetS + RAS groups, which may be consistent with adaptive hyperplasia (Figure 3B,F). The MetS + RAS + MSC group was not different from any other group. Thus, the MSCs reversed maladaptive adipocyte hypertrophy in the perirenal, but not in the subcutaneous fat.

### 3.5. MSCs Reduce Fat Inflammation in MetS + RAS

**Figure 4:**
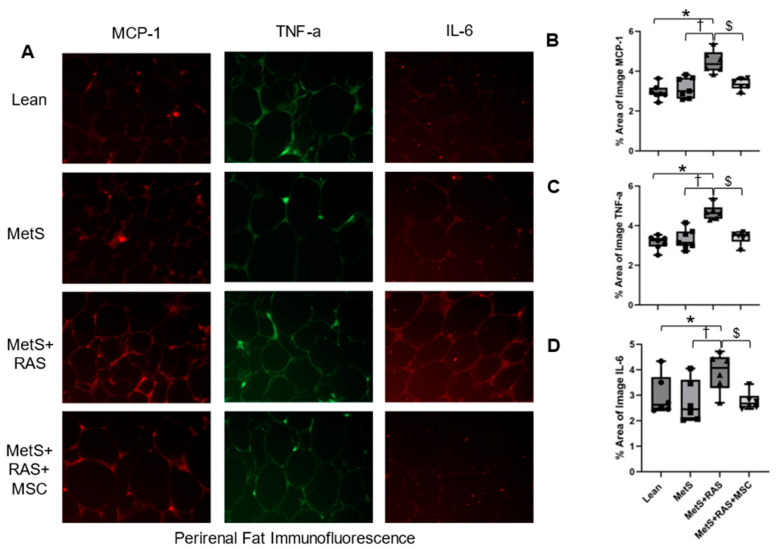
MSCs downregulate perirenal adipose tissue inflammation. (**A**) Representative immunofluorescence images (40×) showing the expression of MCP-1 (red), TNF-a (green), and IL-6 (red) in pig perirenal fat. MCP-1 (**B**), TNF-a (**C**), and IL-6 (**D**) were upregulated in MetS + RAS relative to Lean and MetS but were downregulated in MetS + RAS + MSC. * *p* < 0.05 vs. Lean; ^†^ *p* < 0.05 vs. MetS; ^$^ *p* < 0.05 vs. MetS + RAS. MetS, metabolic syndrome; RAS, renal artery stenosis; MSC, mesenchymal stem/stromal cell.

**Figure 5:**
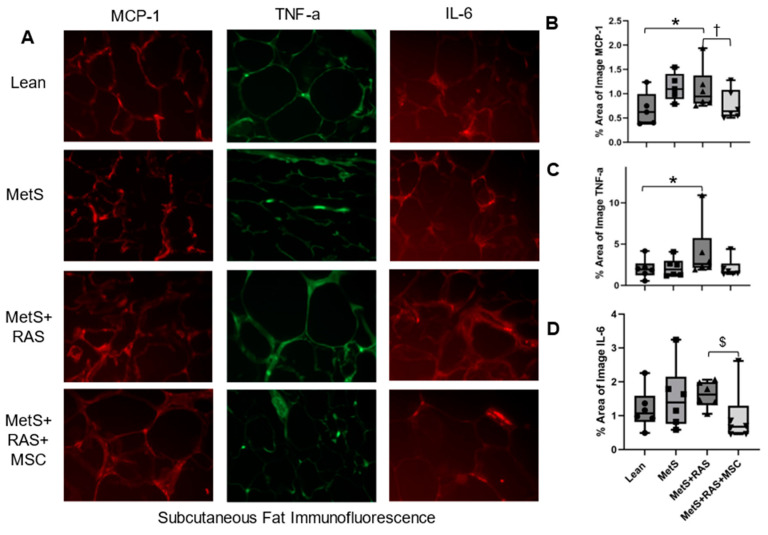
MSCs downregulate subcutaneous adipose tissue inflammation. (**A**) Representative immunofluorescence staining images (40×) showing the expression of MCP-1 (red), TNF-a (green), and IL-6 (red) in pig subcutaneous fat. MCP-1 (**B**) and TNF-a (**C**) were upregulated in MetS + RAS vs. Lean. MCP-1 significantly decreased in MetS + RAS + MSC and TNF-a tended to decrease as well. (**D**) IL-6 expression was not elevated in MetS or MetS + RAS vs. Lean. Nevertheless, MSCs reduced IL-6 expression compared to MetS + RAS. * *p* < 0.05 vs. Lean; ^†^ *p* < 0.05 vs. MetS; ^$^ *p* < 0.05 vs. MetS + RAS. MetS, metabolic syndrome; RAS, renal artery stenosis; MSC, mesenchymal stem/stromal cell.

We performed immunofluorescent staining of the proinflammatory MCP-1, TNF-a, and IL-6 cytokines to evaluate the impact of MSCs on the expression of these proteins in the perirenal and subcutaneous fat. In the perirenal fat, MCP-1 (Figure 4A,B), TNF-a (Figure 4A,C), and IL-6 (Figure 4A,D) were all upregulated in the MetS + RAS group compared to the Lean and MetS groups but decreased in the MetS + RAS + MSC group relative to the MetS + RAS group (Figure 4). In the subcutaneous fat, MCP-1 and TNF-a immunoreactivity was upregulated in the MetS + RAS group vs. the Lean group but normalized in the MetS + RAS + MSC group (Figure 5A–C). IL-6 protein subcutaneous immunoreactivity was not upregulated in any group but was decreased in the MetS + RAS + MSC group relative to the MetS + RAS group (Figure 5D).

**Figure 6:**
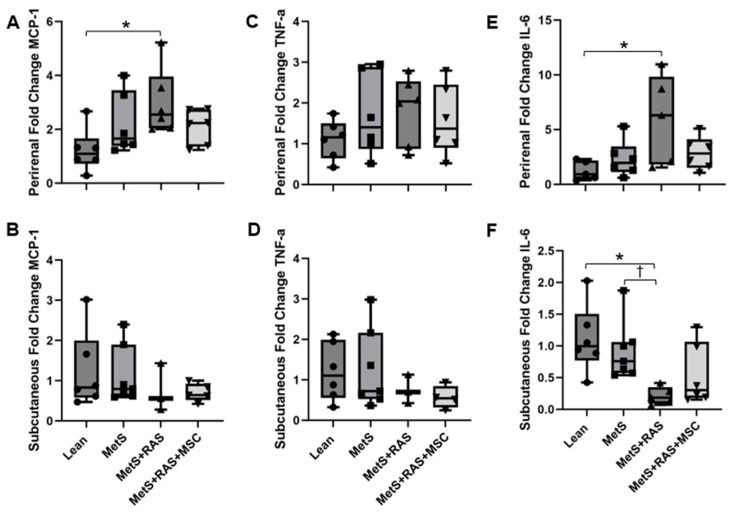
MSCs downregulate proinflammatory cytokine gene expression. (**A**,**C**,**E**) MCP-1 and IL-6 gene expression was upregulated in the MetS + RAS perirenal fat relative to Lean, but MSC treatment attenuated this effect. A slight elevation of TNF-a gene expression (*p* = 0.09) in the perirenal fat of MetS + RAS did not achieve statistical significance. (**B**,**D**,**F**) In the subcutaneous fat, no differences in MCP-1 or TNF-a gene expression were observed among the four groups. However, IL-6 mRNA expression was reduced in the MetS + RAS pigs, while in the MetS + RAS + MSC group, IL-6 levels trended lower relative to the Lean group (*p* = 0.08) but did not reach statistical significance. * *p* < 0.05 vs. Lean; ^†^ *p* < 0.05 vs. MetS. MetS, metabolic syndrome; RAS, renal artery stenosis; MSC, mesenchymal stem cells.

We also performed PCR to evaluate the mRNA expression of these cytokines in the perirenal and subcutaneous fat. In the perirenal fat of the MetS + RAS group, MCP-1 and IL-6 mRNA expression levels were elevated vs. the Lean group but normalized with MSC treatment (Figure 6A,E). Perirenal fat TNF-a tended to be elevated in the MetS + RAS group vs. the Lean group but did not achieve statistical significance (*p* = 0.09, Figure 6C). In contrast, in the subcutaneous fat, no statistically significant differences in the MCP-1 and TNF-a mRNA expression levels were observed among the four groups (Figure 6B,D). However, IL-6 mRNA expression in the subcutaneous fat of the MetS + RAS group was decreased relative to the Lean and MetS groups (Figure 6F). This decrease was not observed in the MetS + RAS + MSC group.

Therefore, MetS + RAS exhibited amplified inflammation compared to other groups, which was particularly notable in the perirenal fat, and to a lesser extent in the subcutaneous fat. This inflammation was preferentially attenuated in the perirenal fat by the intrarenal MSCs delivery.

**Figure 7:**
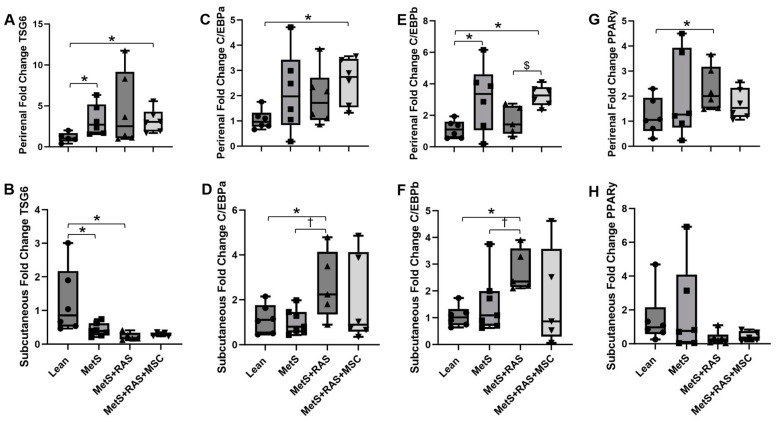
MSCs alter perirenal and subcutaneous anti-inflammatory and adipogenic gene expression. (**A**) Perirenal fat showed upregulated TSG-6 expression in MetS and MetS + RAS + MSC relative to Lean. (**B**) In contrast, subcutaneous fat showed downregulated TSG-6 expression in MetS and MetS + RAS relative to Lean. MetS + RAS + MSC tended to be lower than Lean, but this did not reach statistical significance (*p* = 0.06). C/EBPa expression increased only in the perirenal fat of MetS + RAS + MSC pigs relative to Lean (**C**) and in the subcutaneous fat of MetS + RAS relative to Lean and MetS (**D**). (**E**) Perirenal fat C/EBPb expression was upregulated in MetS relative to Lean and in MetS + RAS + MSC relative to Lean and MetS + RAS. (**F**) Subcutaneous fat expression of C/EBPb was upregulated in MetS + RAS relative to Lean and MetS. PPARy was upregulated in the perirenal fat of MetS + RAS relative to Lean (**G**) but was unchanged in the subcutaneous fat of all pig groups (**H**). * *p* < 0.05 vs. Lean; ^†^ *p* < 0.05 vs. MetS; ^$^ *p* < 0.05 vs. MetS + RAS. MetS, metabolic syndrome; RAS, renal artery stenosis; MSC, mesenchymal stem cells.

The anti-inflammatory protein TSG-6 is a key vector through which MSCs attenuate inflammation [22]. TSG-6 mRNA expression was upregulated in the perirenal fat of the MetS and the MetS + RAS + MSC groups, but not in the MetS + RAS group (Figure 7A). In contrast, it was downregulated in the subcutaneous fat of the MetS and the MetS + RAS groups relative to the Lean group and tended to be lower in the MetS + RAS + MSC group as well (*p* = 0.06 vs. Lean, Figure 7B). TSG-6 upregulation in the perirenal fat of MetS pigs might represent a compensatory mechanism for inflammatory changes that were lost with the superimposition of RAS but restored by the MSCs. The intrarenal delivery of MSCs in the MetS + RAS + MSC group did not restore TSG-6 expression in the subcutaneous fat.

### 3.6. MSCs Alter Adipogenic Gene Expression in Perirenal Adipose Tissue

The transcription factors C/EBPa and C/EBPb regulate the differentiation of pre-adipocytes into mature adipocytes primarily through upregulating PPARy [4]. Interestingly, PPARy, but neither C/EBP-alpha nor beta were upregulated in the perirenal adipose tissue of MetS + RAS pigs relative to Lean pigs (Figure 7C,E,G). This may indicate a relatively late stage of preadipocyte differentiation. MSCs reversed this expression pattern in the MetS + RAS pigs. In contrast, in the subcutaneous fat of MetS + RAS + MSC, both C/EBP-alpha and beta were upregulated relative to Lean, whereas PPARy was not (Figure 7D,F,H). This may indicate an earlier stage in the preadipocyte differentiation process. MSCs downregulated C/EBP-alpha and beta in MetS + RAS but did not affect PPARy expression.

## 4. Discussion

The principal finding of this study is that intrarenal injection of MSCs reverses the maladaptive remodeling primarily observed in the perirenal and, to some extent, the subcutaneous fat of pigs with MetS + RAS. Specifically, MSCs reduced inflammatory cytokine expression in both perirenal and subcutaneous fat (particularly the former), decreased adipocyte size and fibrosis, and mediated a gene expression profile consistent with less advanced fat remodeling. These beneficial effects may be due to a reduction in renal inflammation.

Our MetS and MetS + RAS pig models showed significant weight gain, hypertension, and hypercholesterolemia, although insulin resistance was not observed in this study. However, the HOMA-IR was decreased in the MetS + RAS + MSC group, indicating that MSCs blunted insulin resistance without affecting cholesterol metabolism. In our early model of MetS with unilateral RAS, SCr was not affected. PRA was significantly elevated in all experimental groups, whereas MAP was elevated only in the MetS and MetS + RAS groups compared to the Lean group, but not in the MetS + RAS + MSC group, possibly due to high variability. Thus, MSCs likely exert their primary effects through mechanisms other than the RAAS. Furthermore, MSCs blunted renal inflammation. Both the MCP-1 and TNF-a levels were elevated in the renal vein effluent of the MetS + RAS group compared to the Lean group, which was attenuated by MSCs. These effects were not observed in the systemic circulation, suggesting that the MSCs primarily exerted their effects locally, consistent with other studies [33].

Abdominal CT scan analysis revealed that the subcutaneous fat was significantly expanded in all experimental groups. However, the visceral fat volume, which was expanded in the MetS and MetS + RAS groups vs. the Lean group, was less increased in the MetS + RAS + MSC group. This suggested that the MSCs selectively blunted visceral fat expansion. Therefore, we further characterized and compared their effects on the structure, inflammatory microenvironment, and gene expression of both perirenal and subcutaneous fat.

MetS causes maladaptive adipose hypertrophy and inflammation, leading to fibrosis. Interestingly, we observed that the perirenal adipocyte size was increased only in the MetS + RAS group compared to the Lean group, suggesting that RAS and RVH exacerbated perirenal fat remodeling, which was significantly reduced by the MSC treatment. The subcutaneous adipocyte size showed a different remodeling pattern, with smaller adipocytes in the MetS group compared to the Lean group, consistent with adipocyte hyperplasia, which might be a beneficial adaptation [34] that is impaired in hypertrophic obesity [35]. Alternatively, the observation of adipocyte hyperplasia may also be related to the relatively young age of our juvenile pig model. The MetS + RAS group had larger subcutaneous adipocytes compared to the MetS group, but not compared to the Lean group. However, the MetS + RAS + MSC group did not differ in subcutaneous fat remodeling from the other groups, indicating that the chief effects of the MSCs were in the perirenal fat.

Fibrosis in the perirenal and subcutaneous fat followed a similar pattern. Perirenal fat fibrosis was highest in the MetS + RAS group compared to all other groups but was attenuated in the MetS + RAS + MSC group. Fat fibrosis was less pronounced in the subcutaneous fat, was elevated only in the MetS + RAS group compared to the MetS group, and was unaffected by MetS + RAS + MSC. Therefore, fat fibrosis and hypertrophy were amplified in the MetS + RAS perirenal fat but were attenuated by the MSCs, whereas both fat remodeling and the impact of MSCs were less notable in the subcutaneous fat.

Using immunofluorescence staining, we found that the protein expression of the inflammatory markers MCP-1, TNF-a, and IL-6 [36,37] was upregulated in the perirenal fat of only MetS + RAS compared to other groups, suggesting that MSCs may have blunted perirenal fat inflammation. A similar but less pronounced effect was observed in the subcutaneous fat: MCP-1 and TNF-a were both upregulated in the MetS + RAS group compared to the Lean group, but not in the MetS + RAS + MSC group. Moreover, although IL-6 immunoreactivity was not upregulated in the MetS + RAS group vs. the Lean group, it was significantly lower in the MetS + RAS + MSC group compared to the MetS + RAS group.

We also found that in the perirenal fat, the gene expression of MCP-1 and IL-6 followed a similar pattern to their protein expression, with upregulation in the untreated but not in the MSC-treated MetS + RAS. TNF-a gene expression also tended to increase in MetS + RAS. Interestingly, in the subcutaneous fat, MCP-1 and TNF-a mRNA expression remained unchanged, whereas IL-6 mRNA was decreased in the MetS + RAS group compared to the Lean and MetS groups. Disparities between gene and protein expression are not infrequently encountered and might be related to different methods (staining vs. PCR) or regulation of protein translation and degradation. The observed downregulation of IL-6 in the subcutaneous but not the perirenal fat of the MetS + RAS group may be secondary to the depot-dependence of IL-6 release [38], as both its release and activity differ between visceral and subcutaneous fat [39]. IL-6 release by a tissue is regulated by metabolic stress and exercise, and the sedentary nature of a MetS + RAS treatment may not be conducive to its production in subcutaneous fat. On the other hand, obesity selectively increases IL-6 production, predominantly from visceral fat [40]. However, downregulation of IL-6 in the subcutaneous fat was no longer detected in the MetS + RAS + MSC group. This further confirmed the regional impact of intrarenal artery-injected MSCs on the perirenal fat relative to the subcutaneous fat.

To further explore the effects of MSC on adipose remodeling, we evaluated the gene expression of C/EBPa, C/EBPb, and PPARy due to their involvement in this process [4]. C/EBPa and C/EBPb primarily function through upregulating PPARy. Interestingly, PPARy was elevated only in the perirenal fat of the MetS + RAS group, whereas C/EBPa and C/EBPb were contrarily elevated in the perirenal fat of the MetS + RAS + MSC group, as well as in the subcutaneous fat of th eMetS + RAS group. MSC-mediated upregulation of C/EBPa and C/EBPb, but not PPARy, may represent an earlier phase in adipogenesis. In addition, their upregulation might have been secondary to the direct downregulation of PPARy [41] by MSCs. Thus, by regulating these genes, MSCs may have delayed adipocyte hypertrophy in MetS + RAS pigs. On the other hand, C/EBP alpha and beta, but not PPARy, were upregulated in the subcutaneous fat of the MetS + RAS group and were decreased by MSCs.

TSG-6 is a primary vehicle through which MSCs exert their anti-inflammatory properties [22], and we found it to be upregulated in the perirenal fat of th eMetS group, likely a compensatory response to inflammation, which was blunted in the MetS + RAS group but restored by MSCs. In the subcutaneous fat, TSG-6 was significantly decreased in the MetS and the MetS + RAS groups, suggesting that this depot lacks this compensatory response or is equipped with alternative anti-inflammatory mechanisms. The decrease did not achieve statistical significance in the MetS + RAS + MSC group, suggesting a subtle improvement in the anti-inflammatory microenvironment of the perirenal fat compared to the subcutaneous fat.

The mechanisms by which intrarenal MSCs impacted the perirenal fat remain to be explored. The perirenal fat is a metabolically active tissue that employs paracrine or endocrine mechanisms to regulate kidney homeostasis [42] and serves as a reservoir of MSCs, which resemble those harvested from other fat depots [43]. On the other hand, a retrograde regulation of perirenal fat by changes within the kidney is less well understood. The presence of renal cell carcinoma is associated with altered perirenal fat signaling [44], but the cause and effect remain unclear. Further studies are needed to determine if the kidney regulates perirenal fat through local or systemic pathways, and whether similar effects are exerted on other visceral fat depots.

The limitations of our study include the use of young animals with a short disease duration and potentially age-specific regulation of fat development. However, the strengths of this investigation lie in the human-like features of the pig model, such as similar renal injury and activation of the RAAS observed in humans. The reduction in systemic and fat tissue inflammatory cytokines, along with the increase in TSG-6, supports the anti-inflammatory effects of MSCs. However, we cannot entirely exclude the possibility that some of these effects were also due to the modest decrease in blood pressure compared to the MetS and MetS + RAS group.

Additionally, the autologous MSCs used in this study were harvested after only six weeks of MetS, a stage at which the fat tissue remains relatively healthy [45], and were therefore likely still functional. Further research is needed to assess the efficacy of MSCs obtained after a longer disease period. Future studies should further address the differences in local vs. systemic MSC injections, as well as single vs. multiple injections to determine optimal dosing strategies.

Our study suggests that injecting MSCs into the stenotic renal artery can reduce local inflammation and, to a lesser extent, systemic inflammation and blood pressure. It may also have a direct effect on metabolically active adipose tissue. These findings could guide future research by focusing on depot-specific fat remodeling. Additional studies are necessary to determine the optimal dose and timing of cell delivery, as well as the long-term benefits of cell treatment. Nevertheless, the short-term improvements in systemic inflammatory markers, adipose tissue cellular composition, structure, and gene expression are promising.

## 5. Conclusions

Intrarenal delivery of MSCs into pigs with concurrent metabolic syndrome and renal artery stenosis attenuated the pro-inflammatory phenotype associated with MetS + RAS treatment. It improved fat tissue remodeling, reduced pro-inflammatory cytokine expression, reduced adipocyte fibrosis and size, and modulated a beneficial gene expression profile. These effects are particularly pronounced in the perirenal fat, which is part of the visceral fat depot. Further clinical studies are needed to evaluate the efficacy of injecting autologous MSCs into human patients.

## Figures and Tables

**Table 1:** Systemic characteristics and single-kidney function in study groups at 16 weeks.

	Lean (*n* = 6)	MetS (*n* = 6)	MetS + RAS (*n* = 6)	MetS + RAS + MSC (*n* = 6)
Body Weight (kg)	71 ± 12	94 ± 2 *	92 ± 7 *	81 ± 14 ^†^
Stenosis (%)	0	0	60 ± 24 *	88 ± 19 *^$^
Fasting Glucose (mg/dL)	146 ± 16	157 ± 42	150 ± 37	130 ± 13
Fasting Insulin (ulU/mL)	24 ± 4.2	27 ± 6.2	33 ± 11	22 ± 4.7
HOMA-IR	8.5 ± 1.1	10 ± 2.5	12 ± 4.0	7.2 ± 1.9 ^$^
Total Cholesterol (mg/dL)	75 ± 7	434 ± 219 *	417 ± 142 *	479 ± 285 *
SCr (μmol/L)	1.60 ± 0.31	1.79 ± 0.15	1.73 ± 0.18	1.93 ± 0.21
PRA (ng/mL/h)	98.53 ± 7.21	187.7 ± 59.58 *	185.71 ± 34.00 *	187.40 ± 60.08 *
MAP (mmHg)	103 ± 11	125 ± 9 *	131 ± 18 *	127 ± 39
Renal Vein MCP-1 (pg/mL)	433 ± 395	332 ± 120	2143 ± 1519 *^†^	588 ± 423
Systemic MCP-1 (pg/mL)	147 ± 89	705 ± 413 *	1284 ± 781 *	768 ± 286 *
Renal Vein TNF-a (pg/mL)	36.3 ± 11	97.2 ± 98	63.9 ± 20	36.3 ± 15
Systemic TNF-a (pg/mL)	43.6 ± 22	33.2 ± 7.7	96.1 ± 74	43.4 ± 18

Monocyte chemoattractant protein-1; TNF-a: tumor necrosis factor-alpha. * *p* < 0.05 vs. Lean; ^†^ *p* < 0.05 vs. MetS; ^$^ *p* < 0.05 vs. Mets + RAS.

## Data Availability

The data that support this study are available from the corresponding authors upon reasonable request.
